# LINC00673 Represses CDKN2C and Promotes the Proliferation of Esophageal Squamous Cell Carcinoma Cells by EZH2-Mediated H3K27 Trimethylation

**DOI:** 10.3389/fonc.2020.01546

**Published:** 2020-08-18

**Authors:** Menghan Zhou, Yuhang Mao, Shenling Yu, Yiping Li, Rong Yin, Qin Zhang, Tianyu Lu, Rui Sun, Shaofeng Lin, Yanyan Qian, Ying Xu, Hong Fan

**Affiliations:** ^1^The Key Laboratory of Developmental Genes and Human Diseases, Ministry of Education, Department of Medical Genetics and Developmental Biology, Medical School of Southeast University, Nanjing, China; ^2^School of Life Sciences and Technology, Southeast University, Nanjing, China; ^3^Department of Pathology and Pathophysiology, Medical School of Southeast University, Nanjing, China; ^4^Jiangsu Key Laboratory of Molecular and Translational Cancer Research, Department of Thoracic Surgery, Jiangsu Cancer Hospital, Jiangsu Institute of Cancer Research, The Affiliated Cancer Hospital of Nanjing Medical University, Nanjing, China; ^5^Department of Pathology, Fujian Cancer Hospital & Fujian Medical University Cancer Hospital, Fuzhou, China

**Keywords:** ESCC, LINC00673, CDKN2C, H3K27me3, cell cycle

## Abstract

Long non-coding RNAs (lncRNAs), some of the most abundant epigenetic regulators, play an important role in esophageal squamous cell carcinoma (ESCC). In the current study, the functions and mechanisms of the lncRNA LINC00673 were investigated. The expression levels of LINC00673 and its potential target genes were assessed by quantitative real-time polymerase chain reaction (qPCR) in ESCC surgical specimens and ESCC cell lines. RNA fluorescence *in situ* hybridization (RNA FISH) was employed to detect the subcellular location and the levels of LINC00673 in ESCC samples from patients with different survival times. LINC00673 function in ESCC carcinogenesis was also evaluated *in vivo* and *in vitro*. Cell cycle synchronization was performed using serum withdrawal; the cell cycle was monitored by fluorescence analysis and cellular DNA was detected by flow cytometry. The molecular mechanisms underlying LINC00673 were explored *via* Western blotting, chromatin immunoprecipitation (ChIP), and ChIP-PCR. Up-regulated LINC00673 was associated with poor prognosis in ESCC patients and promoted the proliferation of ESCC cells both *in vitro* and *in vivo*. Compared to the control group, depletion of LINC00673 in ESCC cells arrested the cell cycle, at least, at the G1/S checkpoint. Knockdown of LINC00673 significantly enhanced posttranscriptional expression of CDKN2C, and histone 3 lysine 27 trimethylation (H3K27me3) was enriched at the promoter region of *CDKN2C*. After inhibiting EZH2, the CDKN2C expression levels were increased. The present findings are the first to reveal that LINC00673 represses CDKN2C expression and promotes ESCC cell proliferation by elevating EZH2-mediated H3K27me3 levels. These data suggest that LINC00673 regulates the cell cycle in ESCC and that it is a promising target for clinical therapy.

## Introduction

Esophageal cancer (EC) is one of the most prevalent solid tumor cancers worldwide, ranking seventh, and sixth in morbidity and mortality, respectively (1). Notably, the incidence rate in East Asia, especially in Mongolia and China, ranks highest among regions (1). Furthermore, EC subtype incidence exhibits geographic variation; the most common histologic subtype in China is esophageal squamous cell carcinoma (ESCC), whereas the rates of esophageal adenocarcinoma (EAC) are rising rapidly in Europe and America (2, 3). Despite advances in surgical treatments over the past few decades, the poor prognosis of ESCC has not improved significantly (4, 5). Therefore, the mechanisms underlying human ESCC progression need to be further investigated.

Long non-coding RNAs (lncRNAs) are transcripts of more than 200 nucleotides that do not encode a protein due to a lack of an open reading frame. lncRNAs are critical regulators of multiple disease processes and may function as oncogenes or tumor suppressors by modulating gene expression at the epigenetic, transcriptional, and posttranscriptional levels (6–9). For instance, HOTAIR, and MALAT1 are associated with a variety of human cancers and have potential value in patients’ prognosis (10, 11). It is commonly recognized that lncRNAs, which function as signals of specific cellular states, could be used to identify the cytopathology, predict prognosis, or even guide therapeutic options for patients (10, 12). Nonetheless, compared with other solid tumors with a high incidence, a few studies of lncRNAs in ESCC have been reported; furthermore, the molecular mechanisms of lncRNAs are intricate, and they have not been fully elucidated.

Genomic copy number variations (CNVs) and abnormal expression of genes commonly occur in various tumors, including ESCC. Interestingly, the lncRNA LINC00673 is located at 17q24.3, a chromosomal region that has been found to be frequently amplified in ESCC (13, 14). In addition, LINC00673 has been reported to be associated with cell migration, invasion, and proliferation in squamous cell carcinomas, such as non-small cell lung carcinoma (NSCLC) (15–18), and tongue squamous cell carcinoma (TSCC) (19). Therefore, we investigated whether LINC00673 contributes to ESCC. In the present study, we explored the biological functions and molecular epigenetic mechanisms of LINC00673 in ESCC, and our findings indicate that LINC00673 might be a regulator of CDKN2C through histone methylation modification.

## Materials and Methods

### Patients and Tissue Samples

Paired cancerous tissues and adjacent non-cancerous samples were collected from 39 ESCC patients who underwent surgical resection at Jiangsu Cancer Hospital between 2014 and 2016. All ESCC samples were obtained during surgery and then immediately frozen in liquid nitrogen until RNA detection. In addition, 81 archival paraffin-embedded surgical specimen blocks were obtained from patients who underwent surgery for ESCC from 2004 to 2006 at the Department of Pathology, Fujian Province Tumor Hospital, China (Supplementary Table S1). Clinical information was collected, and the 8th version of the American Joint Committee on Cancer (AJCC) system was applied for tumor staging. Our study protocol was approved by the Ethical Committee of Southeast University.

### Cell Culture

The human ESCC cell lines (KYSE30, KYSE510, and EC9706) were purchased from Guangdong Hybribio Biotech Ltd. (Guangdong, China), and an immortalized normal esophageal epithelial cell line (HET-1A) was purchased from Cinoasia Institute (Shanghai, China). KYSE30, KYSE510, EC9706, and HET-1A cells were cultured in Dulbecco’s modified Eagle’s medium (DMEM; Thermo Scientific) supplemented with 10% fetal bovine serum, 100 U/ml penicillin, and 100 μg/ml streptomycin (Invitrogen, Carlsbad, CA, United States) in a humidified incubator at 37°C with 5% CO_2_.

### RNA Fluorescence *in situ* Hybridization and Scoring

Tissues were dehydrated with gradient alcohol after fixation with paraformaldehyde and then embedded in paraffin. Subsequently, the specimen was cut into slices and baked at 62°C for 2 h. The slices were successively incubated in xylene I for 15 min, xylene II for 15 min, anhydrous ethanol I for 5 min, anhydrous ethanol II for 5 min, 85% alcohol for 5 min, and 75% alcohol for 5 min and then washed with diethyl pyrocarbonate (DEPC)-treated water. The slices were boiled in an antigen repair solution for 10–15 min and then cooled to room temperature. Next, the slices were incubated with proteinase K (20 μg/ml) at 37°C and pre-hybridized with a hybridization buffer at 37°C for 1 h and overnight with a hybridization buffer containing the fluorescence *in situ* hybridization (FISH) probe in the dark in a humid chamber. The samples were washed with 2 × SSC for 10 min at 37°C, 1 × SSC for 2 × 5 min at 37°C, and 0.5 × SSC for 10 min at room temperature. The tissues were then incubated with anti-DIG-488 at 37°C for 50 min, and the nuclei were counterstained with 4′,6-diamidino-2-phenylindole (DAPI). Finally, the slices were sealed in fluorescence decay-resistant medium and images were obtained under a Nikon fluorescence microscope.

Tissue staining was observed by two researchers blinded to the specimen identity. When there was a significant disparity in scoring, a third observer was included to reach an agreement. According to the staining density and scope in ESCC tissues, the staining signals were categorized into three levels: 0 = negative; 1 = weak staining; and 2 = intensive staining.

### shRNA and Plasmid Transfections

The sequences of short hairpin RNAs (shRNAs) targeting LINC00673 were cloned into the GV248 vector, which was purchased from GeneChem (Shanghai, China). Lentiviral plasmids were transfected into KYSE30 and KYSE510 cells according to the manufacturer’s instructions. Transfected cells were subsequently selected with puromycin (1 μg/ml) for 2 weeks. Small interfering RNAs (siRNAs) against EZH2, LINC00673, and the corresponding negative controls were synthesized by GenePharma (Shanghai, China). SiRNA transfection of the ESCC cell lines using Lipofectamine 2000 (Invitrogen, Carlsbad, CA, United States) was performed according to the manufacturer’s protocol. The shRNA and siRNA sequences for the specific targets in this study are shown in Supplementary Table S2.

### RNA Extraction and Quantitative Real-Time PCR

Total RNAs from specimens and cells were isolated with a TRIzol reagent (Invitrogen, United States) according to the manufacturer’s instructions. Reverse transcription of 1 μg RNA to cDNA was performed using a PrimeScript^TM^ RT Reagent Kit (Takara, Japan) under standard conditions. quantitative real-time polymerase chain reaction (qPCR) was performed to determine the expression levels of specific genes using SYBR Premix Ex Taq Kit (Takara, Japan), and β-actin was used as an internal control to normalize the data. All experiments were performed with a StepOne Plus system (Applied Biosystems, Foster City, CA, United States), and the primers used are listed in Supplementary Table S3. All data were calculated using the 2^–ΔΔCt^ method, and each sample was detected in triplicate.

### Cell Proliferation Assay

Esophageal squamous cell carcinoma cell proliferation was detected by Cell Counting Kit-8 (CCK8, Dojindo, Japan) according to the manufacturer’s instructions. Approximately 2.5 × 10^3^ cells per well were seeded into 96-well plates, and all cells were adhered after 6 h. After the cells were incubated with 10 μl CCK8 solution at 37°C in the dark for 2 h, cell proliferation was evaluated by absorbance at 450 nm using an iMark Microplate Reader (Bio-Rad, United States).

### Colony Forming Assay

Esophageal squamous cell carcinoma cells were seeded into fresh 6-cm dishes (10^3^ cells/well) to monitor clonal capacity. 10 days later, the cells were fixed with 75% ethanol and incubated with a Crystal violet solution (Beyotime, Shanghai, China) for 20 min at room temperature and the number of colonies per dish was recorded.

### Flow Cytometric Analysis

Esophageal squamous cell carcinoma cells were synchronized by serum starvation, trypsinized, washed twice with phosphate-buffered saline (PBS), and gently resuspended in cold 75% ethanol. Cell cycle distribution was examined by flow cytometry using a FACScan flow cytometer (Becton Dickinson & Co., San Jose, CA, United States).

### Tumor Xenograft Mouse Model

A sample of approximately 5 × 10^6^ ESCC cells in 0.2 ml PBS was injected subcutaneously into 4-week-old female athymic BALB/c nude mice (Model Animal Research Center of Nanjing University, China). Tumor formation was monitored every 3 days starting on the fourth day after injection, and the tumor volume was calculated using the following formula: *V* = 0.5 × *L* × *W*^2^. The mice were euthanized 4 weeks after injections, and the average tumor weights in each group were measured. All procedures were conducted in accordance with the institutional standard guidelines of the Medical School of Southeast University. The animal experiments in this study were approved by the Experimental Animal Ethics Committee of Southeast University.

### Western Blotting

Protein lysates of ESCC cells were separated by 12% sodium dodecyl sulfate polyacrylamide gel electrophoresis (SDS-PAGE), transferred to 0.22-mm NC membranes (Sigma), and incubated with specific antibodies: anti-CDK6, anti-CDK4, anti-CDK2, anti-cyclinD1, anti-cyclinD3, anti-p27, anti-p21, anti-CDKN2C (Abcam, Shanghai, China), anti-EZH2, anti-EED, anti-SUZ12, anti-EZH1, and anti-β-actin (Cell Signaling Technology). The dilution ratio of the primary antibodies was 1:1,000, although anti-β-actin was diluted to 1:8,000 for Western blotting. Protein bands were visualized with Super Signal Chemiluminescence Substrate (Thermo Scientific) and β-actin was used as a control.

### Chromatin Immunoprecipitation and ChIP-PCR

The chromatin immunoprecipitation (ChIP) assay was performed using an EZ-Magna ChIP^TM^ G Immunoprecipitation Kit (Millipore) following the manufacturer’s instructions. ChIP-grade anti-H3K27me3 (CST, United States; dilution ratio, 1:50) and normal mouse IgG (as a negative control; 1 μg per IP) were used for immunoprecipitation. The primers used for amplifying the precipitated DNA fragments are listed in Supplementary Table S3.

### Statistical Analysis

The data were analyzed using the SPSS (Statistical Package for the Social Sciences) statistical program version 17 (SPSS, Inc., Chicago, IL, United States). An independent Student’s *t* test (two-tailed) was performed to determine statistical significance. The correlations between the target genes were examined with the chi-square test. The data are presented as the mean ± SD. *P* < 0.05 was considered statistically significant (^∗^*P* < 0.05, ^∗∗^*P* < 0.01).

## Results

### Up-Regulated LINC00673 Is Associated With Poor Prognosis in ESCC Patients

**FIGURE 1:**
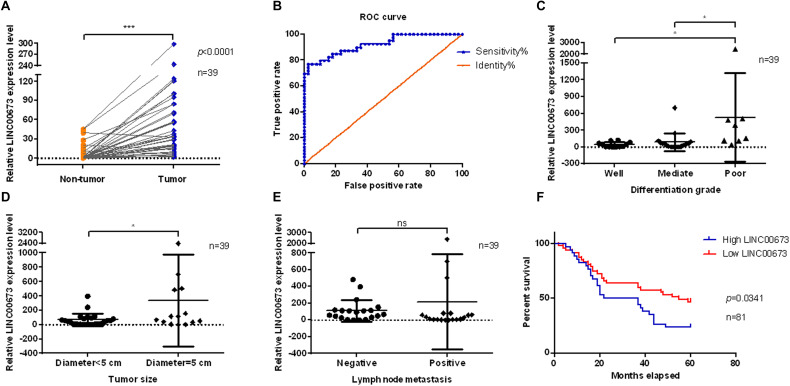
Up-regulated LINC00673 is associated with poor prognosis in esophageal squamous cell carcinoma (ESCC) patients. **(A)** LINC00673 expression levels were evaluated using quantitative PCR (qPCR) in 39 pairs of ESCC tissues and corresponding non-tumor tissues. ***P < 0.001 (unpaired Student’s *t* test). **(B)** Receiver operating characteristic (ROC) curve of LINC00673 in 39 ESCC patients. **(C–E)** Relationships between LINC00673 expression and the differentiation grade, tumor size, and lymph node metastasis, respectively. **(F)** Kaplan–Meier curves show the survival of ESCC patients, as grouped by the LINC00673 expression levels. *P < 0.05.

LINC00673 was evaluated in pairs of tumor tissues and corresponding adjacent non-tumor tissues from 39 ESCC patients using qPCR. LINC00673 showed a pattern of significantly higher expressions in ESCC tissues than in non-tumor tissues (Figure 1A). To confirm this result, we detected LINC00673 expression levels in tissue microarrays with 81 pairs of samples, consisting of ESCC tissues and corresponding adjacent non-tumor tissues, by RNA FISH (Supplementary Figure S1A and Supplementary Table S1). The results of the staining were consistent with the qPCR data (Supplementary Figure S1B). Furthermore, the value of LINC00673 in the diagnosis of ESCC was measured by a receiver operating characteristic (ROC) curve. The results showed a high true positive rate of the LINC00673 expression model for 39 ESCC patients (Figure 1B). In addition, the correlation between the expression levels of LINC00673 and the clinicopathological factors in the 39 ESCC patients was examined with the chi-square test. Up-regulated LINC00673 exhibited a close relationship with poor differentiation and large tumor size, but not with lymph node metastasis (Figures 1C–E). According to the RNA FISH scoring, the LINC00673 expression patterns correlated with differentiation grade, tumor size, and T stage, but not with age, sex, or other clinicopathological features (Table 1). Compared to patients with low LINC00673 levels (score ≤ 1), those with high LINC00673 levels (score > 1) displayed poor outcomes (Figure 1F). This finding suggests that an increased level of LINC00673 expression has a negative impact on the survival of ESCC patients. Taken together, our results demonstrate that LINC00673 is up-regulated in ESCC and can, to some extent, predict poor prognosis.

**TABLE 1:** Correlations between the expression patterns of LINC00673 detected by RNA fluorescence *in situ* hybridization (FISH) and related clinicopathological parameters.

Clinicopathological features	LINC00673 (*n* = 81)			
	Low	High	χ ^2^	*P* value
Sex				
Male	33	27	0.8693	0.3511
Female	14	7		
Age (years)				
≤60	29	23	0.3034	0.5818
>60	18	11		
Tumor sub-sites				
Cervical portion	9	4	0.7984	0.3716
Thoracic portion	38	30		
Differentiation grade				
I + II	40	20	7.096	0.0077**
III	7	14		
Tumor size (maximum diameter)				
≤5 cm	7	12	4.573	0.0325*
>5 cm	40	22		
T stage				
I + II	19	5	6.259	0.0124*
III + IV	28	19		
N stage				
0 + I	31	22	0.01366	0.9069
II + III	16	12		
TNM stage				
I + II	21	16	0.04496	0.8321
III + IV	26	18		

*Significant differences are shown when the *P* value is less than 0.05. **Significant differences are shown when the *P* value is less than 0.01.

### Knockdown of LINC00673 Inhibits ESCC Cell Proliferation *in vitro* and *in vivo*

**FIGURE 2:**
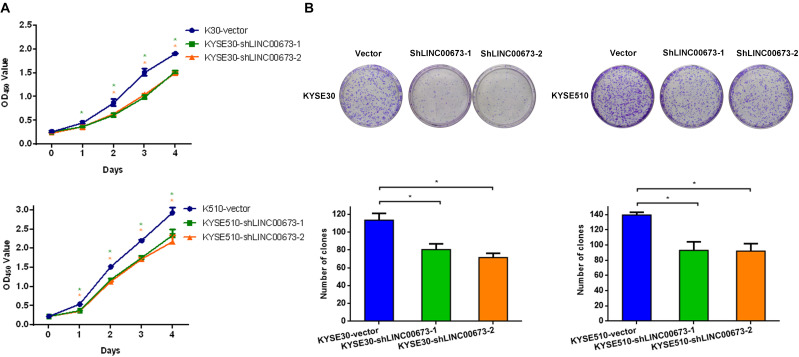
LINC00673 knockdown suppresses the proliferation of esophageal squamous cell carcinoma (ESCC) cells *in vitro*. **(A)** Cell proliferation was assessed by the CCK8 method in KYSE30 and KYSE510 cells. *Bars* show the mean ± SD of OD_450_ from triplicate samples. **(B)** Cell proliferation was assessed using a colony forming assay. *Bar plots* show the average number of colonies (±SD) from triplicate samples. *P < 0.05.

The strong association of LINC00673 expression with the tumor size, differentiation grade, and poor prognosis in ESCC prompted us to investigate whether LINC00673 affects ESCC cell proliferation. Thus, we constructed KYSE30-shLINC00673 cell lines, KYSE510-shLINC00673 cell lines, and corresponding controls and then measured the efficiency of shRNA-LINC00673 (Supplementary Figures S2A,B). Compared to the corresponding controls, the depletion of LINC00673 suppressed the ESCC cell proliferation capacity (Figure 2A). Similarly, the down-regulation of LINC00673 reduced the clone-forming capacity (Figure 2B).

**FIGURE 3:**
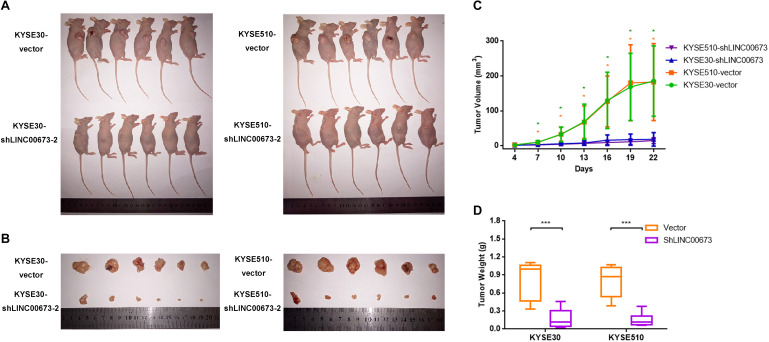
LINC00673 knockdown inhibits esophageal squamous cell carcinoma (ESCC) cell proliferation *in vivo*. **(A,B)** Stable KYSE30-shLINC00673 and KYSE510-shLINC00673 cells were injected subcutaneously into nude mice. After 4 weeks, the tumors were resected. **(C)** Tumor growth was measured by Vernier calipers every 3 days starting on the fourth day after injection. *Line chart* show the tumor growth curve. **(D)** Box plot showing the tumor weights of the control and LINC00673 knockdown groups. *P < 0.05, ***P < 0.001.

To further investigate the role of LINC00673 in the tumorigenesis of ESCC *in vivo*, we performed an *in vivo* tumor formation assay by injecting KYSE30-shLINC00673 cell lines, KYSE510-shLINC00673 cell lines, and their corresponding control cells into BALB/c nude mice (*n* = 6 per group). Tumor growth was measured every 3 days starting on the fourth day after injection, and the mice were euthanized 4 weeks after injection. The results suggested that compared to control cells, smaller tumors developed in mice from cells with stable knockdown of LINC00673 (Figures 3A–C). The tumor weight was also reduced in the LINC00673 knockdown groups compared to the control groups (Figure 3D). Overall, these data suggest that LINC00673 knockdown suppresses ESCC cell proliferation both *in vitro* and *in vivo*.

### Knockdown of LINC00673 Arrests the Cell Cycle at the G1/S Checkpoint in ESCC Cells

**FIGURE 4:**
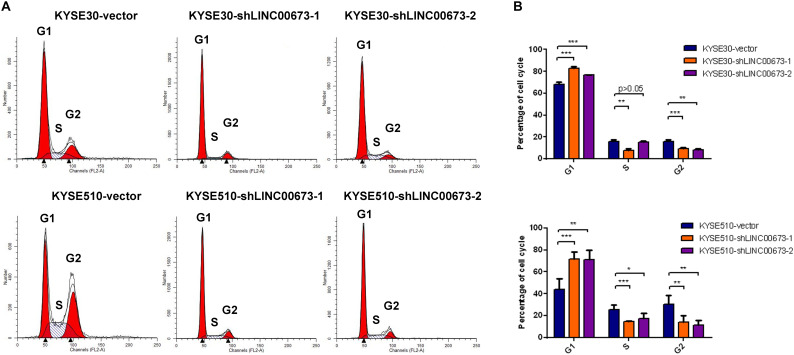
LINC00673 knockdown arrests the cell cycle at the G1/S checkpoint in esophageal squamous cell carcinoma (ESCC) cells. **(A,B)** Cell cycle examined by a flow cytometry assay. *Bar plots* show the proportion of cells in the G1 phase, S phase, and G2 phase. *P < 0.05, **P < 0.01, and ***P < 0.001.

To determine the contribution of the cell cycle to the suppression of ESCC cell proliferation both *in vitro* and *in vivo*, we examined the cell cycle for various expression levels of LINC00673 by flow cytometry. Compared to the control groups, KYSE30 and KYSE510 cells with down-regulated LINC00673 showed higher proportions of cells in the G1 phase (Figures 4A,B), indicating that LINC00673 depletion suppresses cell proliferation by blocking the cell cycle at the G1/S checkpoint. In addition, the relatively low proportions of KYSE30-shLINC00673 and KYSE510-shLINC00673 cells in the G2 phase were shown by the results. It needs further investigation to confirm whether LINC00673 depletion will promote ESCC cells to enter the M phase. In the current study, the data showed that LINC00673 down-regulation inhibited cell proliferation in ESCC; therefore, we preferentially focused on the investigation of G1/S checkpoint arrest rather than the G2/M transition.

**FIGURE 5:**
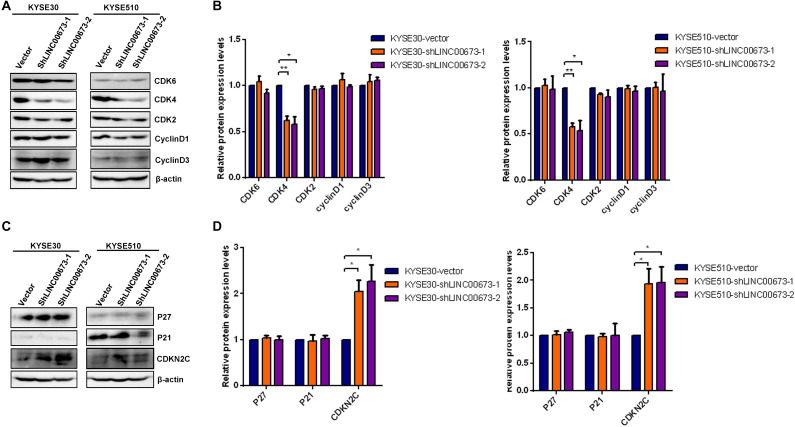
LINC00673 knockdown improved CDKN2C levels in esophageal squamous cell carcinoma (ESCC) cells. **(A,C)** The levels of the G1/S checkpoint regulator expression were detected by Western blotting analysis in stable KYSE30-shLINC00673, KYSE510-shLINC00673, and corresponding control cells. **(B,D)** *Bar plots* showing the relative expression levels of the G1/S checkpoint associated regulators. The image was processed by ImageJ software. *P < 0.05, **P < 0.01.

To determine how LINC00673 regulates cell cycle progression, the expression levels of the G1/S checkpoint regulators CDK2, CDK4, CDK6, cyclin D1, and cyclin D3 were examined by Western blotting. Among these proteins, the expression of CDK4 was significantly down-regulated in the LINC00673 knockdown KYSE30 and KYSE510 cells compared to the control groups (Figures 5A,B). As the CDK inhibitors P27^Kip1^, P21^Cip1^, and CDKN2C are important regulators of the cell cycle (Supplementary Figure S3), we hypothesized that LINC00673 promotes ESCC cell cycle progression by suppressing *p21^Cip1^*, *p27^Kip1^*, or *CDKN2C* expression. As expected, only the CDKN2C protein expression increased when LINC00673 was knocked down in KYSE30 and KYSE510 cells (Figures 5C,D).

Consistent with the depleted level of CDK4, CDKN2C, a cyclin-dependent kinase inhibitor, showed an increasing expression trend compared to the control groups. These findings indicate that LINC00673 knockdown enhances *CDKN2C* expression, further regulating cell cycle progression in ESCC cells.

### H3K27me3 Is Enriched at the Promoter Region of *CDKN2C* to Silence CDKN2C Expression

**FIGURE 6:**
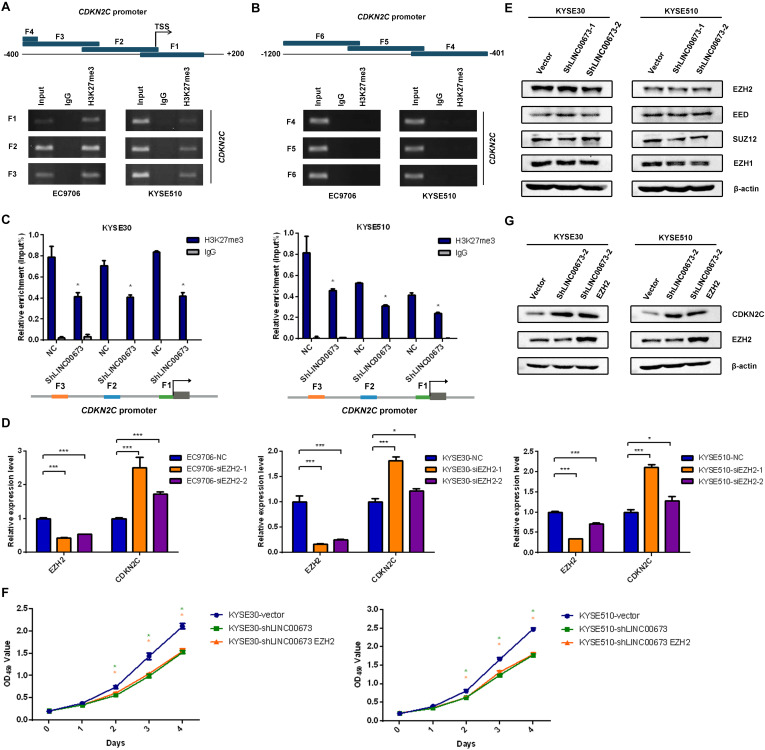
H3K27me3 was enriched at the promoter region of *CDKN2C*. **(A,B)** Diagram of the promoter of the *CDKN2C* gene, with the transcription start site (TSS) indicated. The *blue block* represents the location of the F1–F6 fragments detected by chromatin immunoprecipitation (ChIP). ChIP assays were performed using an H3K27me3-specific antibody, followed by PCR amplification of six individual fragments representing *CDKN2C* promoter regions (*F1*–*F6*). Input and products immunoprecipitated by IgG were used as positive and negative controls, respectively. **(C)** ChIP assays were performed using an H3K27me3-specific antibody in KYSE30-shLINC00673, KYSE510-shLINC00673, and corresponding control cells, followed by quantitative PCR (qPCR) amplification of three individual fragments near the *CDKN2C* promoter (*F1*–*F3*). *P < 0.05 (unpaired Student’s *t* test). **(D)** *CDKN2C* expression levels were detected using qPCR after silencing EZH2 in EC9706, KYSE30, and KYSE510 cells. *P < 0.05, ***P < 0.001. **(E)** The major subunits of the PRC2 complex expression levels were detected by Western blotting analysis in stable KYSE30-shLINC00673, KYSE510-shLINC00673, and corresponding control cells. **(F)** Cell proliferation was assessed by the CCK8 method in KYSE30-shLINC00673 and KYSE510-shLINC00673 cells after transfection with EZH2. *Bars* show the mean ± SD of OD_450_ from triplicate samples. **(G)** EZH2 and CDKN2C expression levels were detected by Western blotting analysis after the transfection of EZH2 in KYSE30-shLINC00673 and KYSE510-shLINC00673 cells.

To clarify whether LINC00673 regulates the cell cycle by inhibiting *CDKN2C*, we conducted a ChIP assay using antibodies against H3K27me3 in EC9706 and KYSE510 cells, as transcriptional repression is characterized by H3K27me3, which serves as a typical suppressor marker associated with inactive gene promoters (20–24). Primers for the *CDKN2C* promoter region were designed to probe DNA fragments *via* PCR (the primers, covering -1,200 to + 150 bp, are shown in Supplementary Table S3). In this assay, the sequences between the *CDKN2C* transcription initiation site (TSS) and the upstream 400 bp were amplified as DNA fragments interacting with H3K27me3 (Figures 6A,B), which indicated that H3K27me3 was indeed enriched at the promoter region of *CDKN2C*. Furthermore, we performed ChIP-qPCR of H3K27me3 in KYSE30-shLINC00673 and KYSE510-shLINC00673 cells and found that the enrichment of H3K27me3 at the *CDKN2C* promoter region decreased in cells with LINC00673 down-regulation compared to the control groups (Figure 6C). This result suggests that LINC00673 regulates CDKN2C expression through H3K27me3.

Several researchers have reported the interaction between LINC00673 and enhancer of zeste 2 polycomb repressive complex 2 subunit (EZH2) in solid tumors (17, 25–27), and EZH2 is generally considered to execute methyltransferase effects against H3K27 (28). Thus, we hypothesized that LINC00673 regulates H3K27me3 levels by recruiting EZH2, affecting the downstream cell cycle regulator CDKN2C. When EZH2 expression was transiently knocked down in three ESCC cell lines—EC9706, KYSE30, and KYSE510—an increased expression of *CDKN2C* was detected, as expected (Figure 6D). In addition, Western blotting was conducted to confirm whether LINC00673 influences the major subunits of the PRC2 complex, and the results showed that the expression levels of the major6 subunits, such as EZH2, SUZ12, EED, and EZH1, were not affected by the LINC00673 expression pattern (Figure 6E and Supplementary Figures S4A,B).

To assess whether EZH2 overexpression can reverse the inhibited cell proliferation and up-regulated CDKN2C levels in LINC00673 knockdown cells, EZH2 pcDNA3.1 plasmids were transfected into KYSE30-shLINC00673 and KYSE510-shLINC00673 cells. Data from CCK8 and the Western blotting assays suggested that up-regulated EZH2 in LINC00673 knockdown cells did not reverse cell proliferation or decrease CDKN2C expression (Figures 6F,G and Supplementary Figures S4C,D), indicating that CDKN2C was regulated by histone modification due to a decrease in EZH2 recruitment rather than a decrease in EZH2 expression. Our hypothesis is that LINC00673 can elevate H3K27me3 levels at the *CDKN2C* promoter by recruiting EZH2. However, due to the knockdown of LINC00673, EZH2 cannot become massively enriched near the *CDKN2C* promoter. Therefore, cell proliferation in KYSE30-shLINC00673 and KYSE510-shLINC00673 cells was not reversed by the overexpression of EZH2. In general, these data show that LINC00673 recruits EZH2 to regulate H3K27me3 levels at the *CDKN2C* promoter region and further modulates the ESCC cell cycle and proliferation.

## Discussion

Esophageal squamous cell carcinoma is characterized by striking geographic variation, intricate etiology, and poor clinical outcomes in China. Various pathogenic factors, such as dietary habits and chemical carcinogens, might cause genetic mutations and epigenetic variations, yet little is known about the epigenetic regulatory mechanisms underlying ESCC progression (29). To improve prognosis and better understand this complicated disease, this study was designed to elucidate the molecular regulatory mechanisms involved in ESCC development.

Chemical modifications of the fundamental macromolecule DNA and proteins participate in the control and adaptability of almost all biological processes. Methylation, known as one of the most prevalent modifications, is widespread throughout all forms of life (30), and many tumor suppressor genes are silenced by DNA methylation in cancers (31). For example, hypomethylation of repetitive elements in mammals can induce the activation and transposition of endogenous retroviral elements, thus promoting instability in the genome and tumorigenesis (30, 32). Histone methylation levels in facultative heterochromatin are also associated with genes that are differentially expressed during cell differentiation or development (30, 33), as characterized by gene-inactive H3K27me3 modification. Developmental genes contain so-called bivalent domains that possess both gene-active H3K4me3 and gene-repressive H3K27me3 modifications. This “poised” state has been shown to allow these genes to respond to differentiation cues quickly and accurately (30, 34). Furthermore, histone methylation, as represented by H3K27me3, H3K9me3, and H4K20me3, is related to the outcome of ESCC patients (23, 35). This evidence suggests that epigenetic modifications, such as methylation, may play an important role in tumor progression.

**FIGURE 7:**
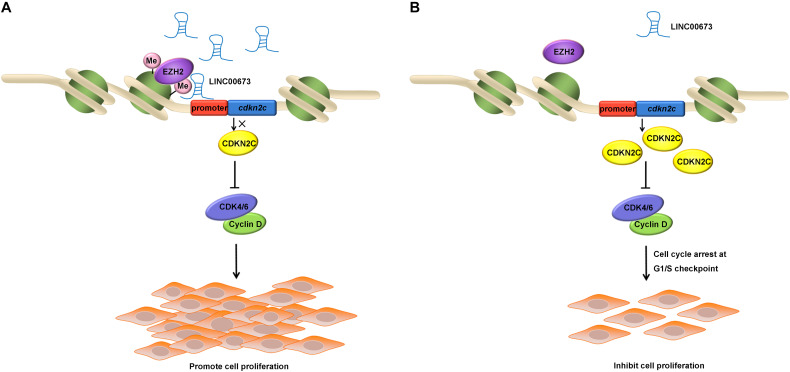
Schematic of the regulatory relationships among LINC00673, EZH2, and CDKN2C in ESCC cells. **(A,B)** Illustrated the effect of up-regulated or down-regulated LINC00673 on ESCC cell proliferation, respectively.

After confirming that depleted LINC00673 was able to up-regulate CDKN2C and promote the cell cycle, we conducted a ChIP assay using antibodies against H3K27me3 in EC9706 and KYSE510 cells and found that H3K27me3 was enriched at the promoter region of *CDKN2C*. Some key molecules that may regulate *CDKN2C* expression *via* methylation modification—DNMT1, DNMT3A, and EZH2 (36–38)—were knocked down in ESCC cells; among these molecules, only EZH2 knockdown could up-regulate CDKN2C. It has been reported that EZH2 recruits LINC00673 (17, 25–27), and it is reasonable to consider that EZH2 is recruited to the *CDKN2C* promoter by LINC00673, increasing the level of H3K27me3 to regulate *CDKN2C* expression and, thus, affecting the proliferation of ESCC cells (Figures 7A,B).

An interesting phenomenon was noted in our study: compared with the control group, ESCC cells with LINC00673 down-regulation had a very dramatic inhibitory effect on tumor formation *in vivo*, whereas this repressive effect of decreased LINC00673 on tumor cell proliferation was not that obvious *in vitro*. These results suggest that the tumor microenvironment may be responsible for the difference between *in vivo* and *in vitro* results. In fact, information exchange in the tumor microenvironment can significantly affect tumor progression and malignant biological behaviors (39–41). Moreover, lncRNAs secreted by tumor-derived exosomes can alter the cellular physiology of cancer cells by elevating their expressions of membrane molecules and soluble factors (42, 43).

In conclusion, our study confirmed that LINC00673 plays a role in promoting tumor proliferation by regulating G1/S checkpoint regulator expression. Furthermore, our data demonstrate that LINC00673 acts as a scaffold protein that recruits EZH2 to repress CDKN2C levels in ESCC. Therefore, LINC00673 may be a promising molecular target for the treatment of ESCC.

## Data Availability Statement

All datasets generated or analyzed in this study are included in the article/Supplementary Material.

## Ethics Statement

Our study protocol was approved by the Ethical Committee of Southeast University. The patients provided their written informed consent to participate in this study.

## Author Contributions

HF conceived and designed the experiments. MZ conducted the experiments and wrote the manuscript. YL, YM, SY, and YX analyzed the experiment data. RY, SL, and QZ provided the ESCC clinical specimens. YQ performed the animal experiment. TL and RS analyzed the RNA FISH data. All authors reviewed the manuscript before submission.

## Conflict of Interest

The authors declare that the research was conducted in the absence of any commercial or financial relationships that could be construed as a potential conflict of interest.
