# Supplementary material for: LINC00673 Represses CDKN2C and Promotes the Proliferation of Esophageal Squamous Cell Carcinoma Cells by EZH2-Mediated H3K27 Trimethylation
Source: Front Oncol. 2020 Aug 18;10:1546. doi: 10.3389/fonc.2020.01546 (PMC7461945; doi:10.3389/fonc.2020.01546)
Supplement: Supplementary file 1 [file Data_Sheet_1.docx]

**Table S1** Clinical and pathological characteristics of ESCC patients (n=81)

| Characteristic | No. | Percentage (%) |
| --- | --- | --- |
| 1. Sex |  |  |
| Male | 60 | 74.1 |
| Female | 21 | 25.9 |
| 2. Age (y) |  |  |
| Age≤60 | 52 | 64.2 |
| Age>60 | 29 | 35.8 |
| 3. Tumor subsites |  |  |
| Cervical portion | 13 | 16.0 |
| Thoracic portion | 68 | 83.9 |
| 4. Differentiation grade |  |  |
| I | 6 | 7.4 |
| II | 54 | 66.7 |
| III | 21 | 25.9 |
| 5. Tumor size (the maximum diameter) |  |  |
| ≤6cm | 74 | 91.4 |
| >6cm | 7 | 8.6 |
| 6. T stage |  |  |
| Ⅰ | 6 | 7.4 |
| Ⅱ | 18 | 22.2 |
| Ⅲ | 54 | 66.7 |
| Ⅳ | 3 | 3.7 |
| 7. N stage |  |  |
| 0 | 27 | 33.3 |
| Ⅰ | 26 | 32.1 |
| Ⅱ | 22 | 27.2 |
| Ⅲ | 6 | 7.4 |
| 8. TNM stage |  |  |
| Ⅰ | 6 | 7.4 |
| Ⅱ | 31 | 38.3 |
| Ⅲ | 44 | 54.3 |

**Table S2** The sequences of siRNAs and shRNAs

| SiRNA and shRNAs | Sequences 5’-3’ |
| --- | --- |
| SiRNAs |  |
| siLINC00673-1  siLINC00673-2  siEzh2-1  siEzh2-2  siDnmt1  siDnmtA | CAGCCGGAUACAGAGUGAAUAGUUATT  UGUGCCUUUGUACUCAGCAAUUCUUTT  AAGACUCUGAAUGCAGUUGCUTT  CGGCUUCCCAAUAACAGUATT  CAAUGAGACUGACAUCAAATT  GCCUCAGAGCUAUUACCCATT |
| ShRNAs |  |
| shLINC00673-1 | CAGCCGGAUACAGAGUGAAUAGUUA |
| shLINC00673-2 | UGUGCCUUUGUACUCAGCAAUUCUU |

**Table S3** Primers used in the present study

| Primers | Sequences 5’-3’ |
| --- | --- |
| Primers for q-PCR |  |
| β-actin sense  β-actin antisense  LINC00673 sense  LINC00673 antisense | GTCATTCCAAATATGAGATGCGT  GCTATCACCTCCCCTGTGTG  TACCACACCCTTTCTTGCCC  ACACTGGCCTCTTTACACGG |
| Ezh2 sense  Ezh2 antisense | TTGTTGGCGGAAGCGTGTAAAATC  TCCCTAGTCCCGCGCAATGAGC |
| Cdkn2c sense  Cdkn2c antisense | CGTCAATGCACAAAATGGATTTGG  GAATGACAGCGAAACCAGTTCG |
| Primers for ChIP-PCR |  |
| Cdkn2c F1 sense  Cdkn2c F1 antisense | GAGCGTGCGAGACTGCGAGC  CTGCTTCTGTTGCCTCTC |
| Cdkn2c F2 sense  Cdkn2c F2 antisense | TCACAGACTCAAGCCCGCCA  TTAAGGAGGCTCGGCAGA |
| Cdkn2c F3 sense  Cdkn2c F3 antisense | GAACGACTCCCTTTATGC  TCTCCACCTCCTCCCGTCAA |
| Cdkn2c F4 sense  Cdkn2c F4 antisense | CATAAAATTCAGTCTACACA  TAAGACGGTTGTGGAGGGC |
| Cdkn2c F5 sense  Cdkn2c F5 antisense | ATTGGTGTCGGATGATTA  GACTGCCACTAAGCACAG |
| Cdkn2c F6 sense  Cdkn2c F6 antisense | TGTTGCTAATACTCCCAG  TTAGAAGTGGCTTGGATT |
