# Supplementary figures and images for: LINC00673 Represses CDKN2C and Promotes the Proliferation of Esophageal Squamous Cell Carcinoma Cells by EZH2-Mediated H3K27 Trimethylation
Source: Front Oncol. 2020 Aug 18;10:1546. doi: 10.3389/fonc.2020.01546 (PMC7461945; doi:10.3389/fonc.2020.01546)

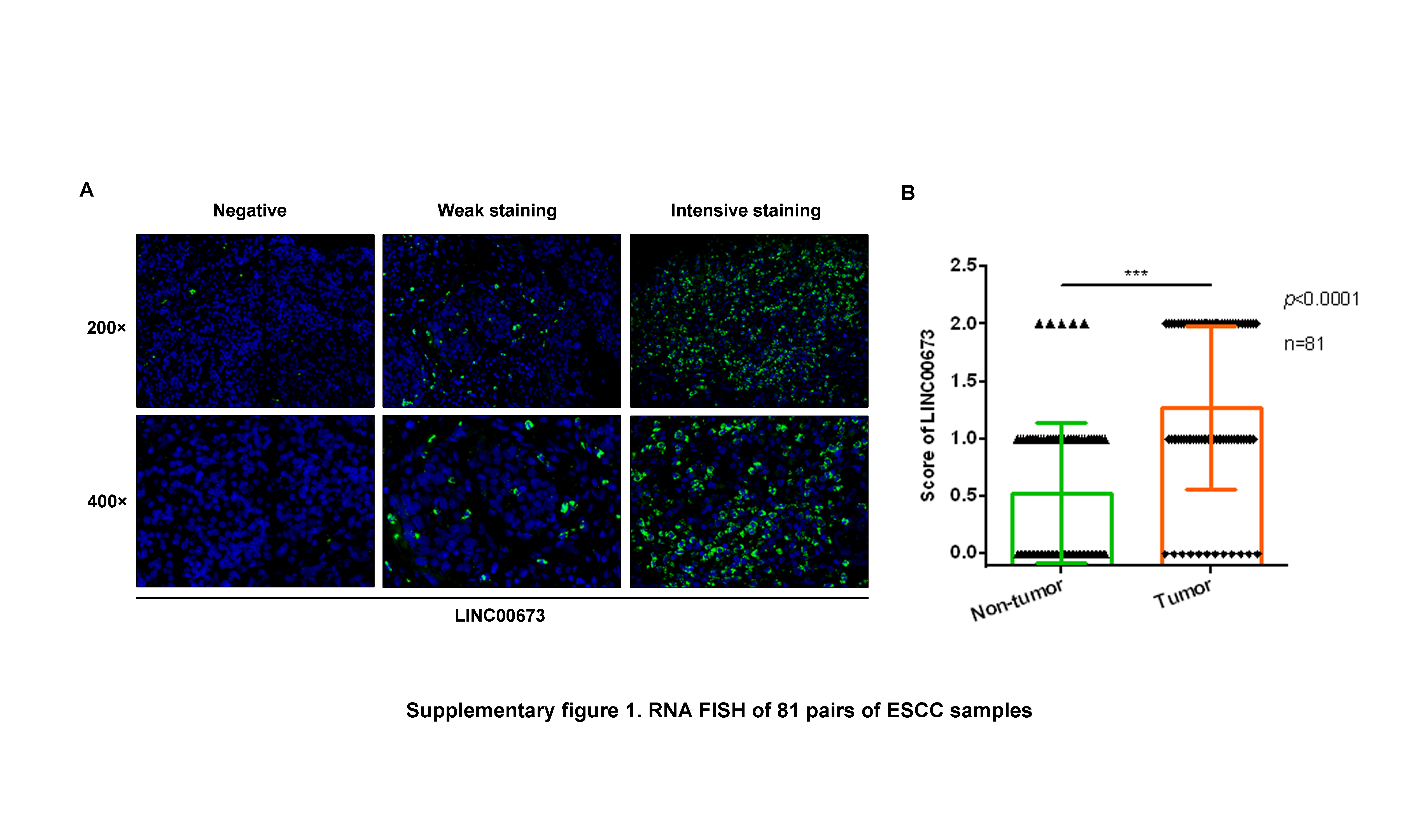

Supplement: Supplementary file 2 [file Image_1.TIF]

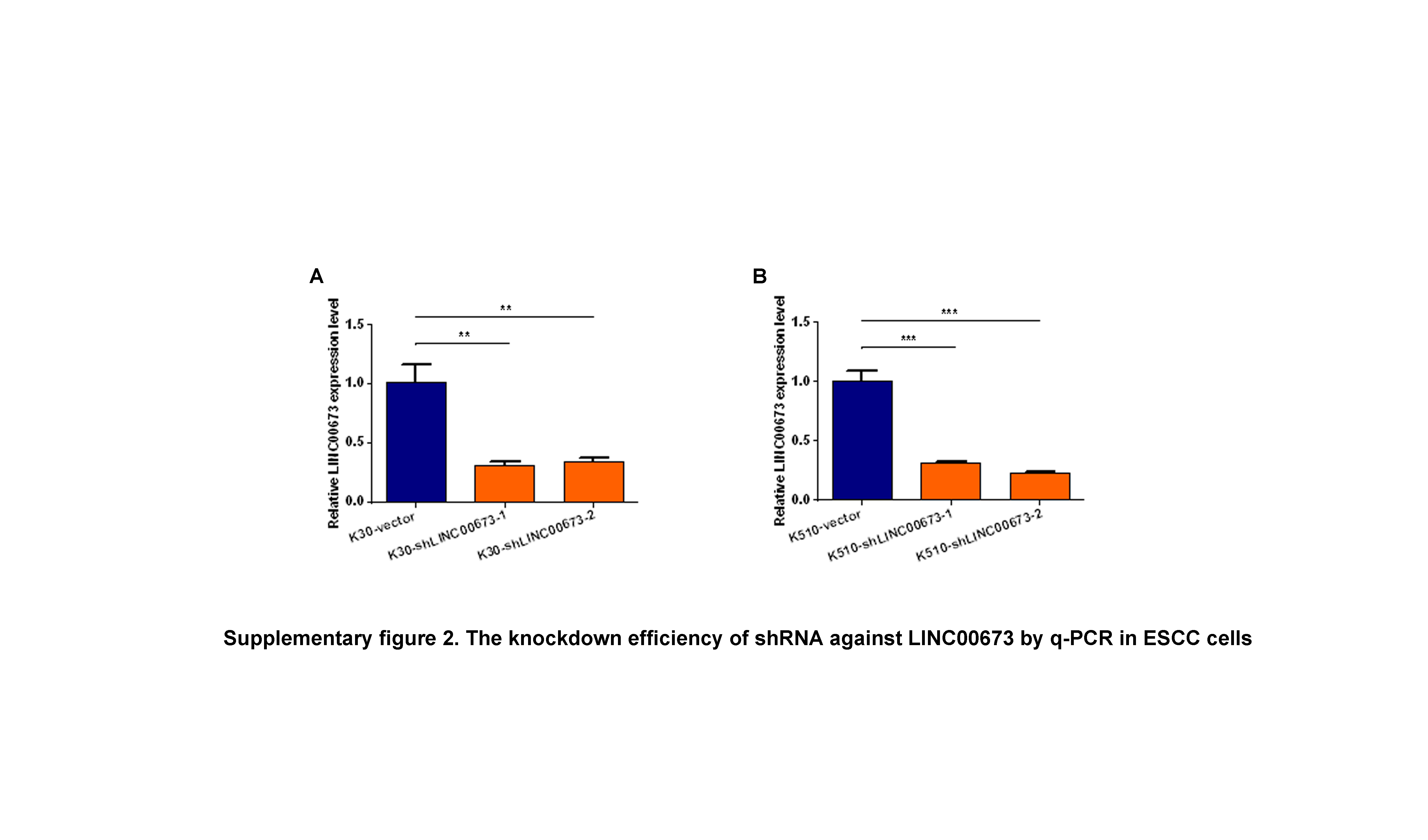

Supplement: Supplementary file 3 [file Image_2.TIF]

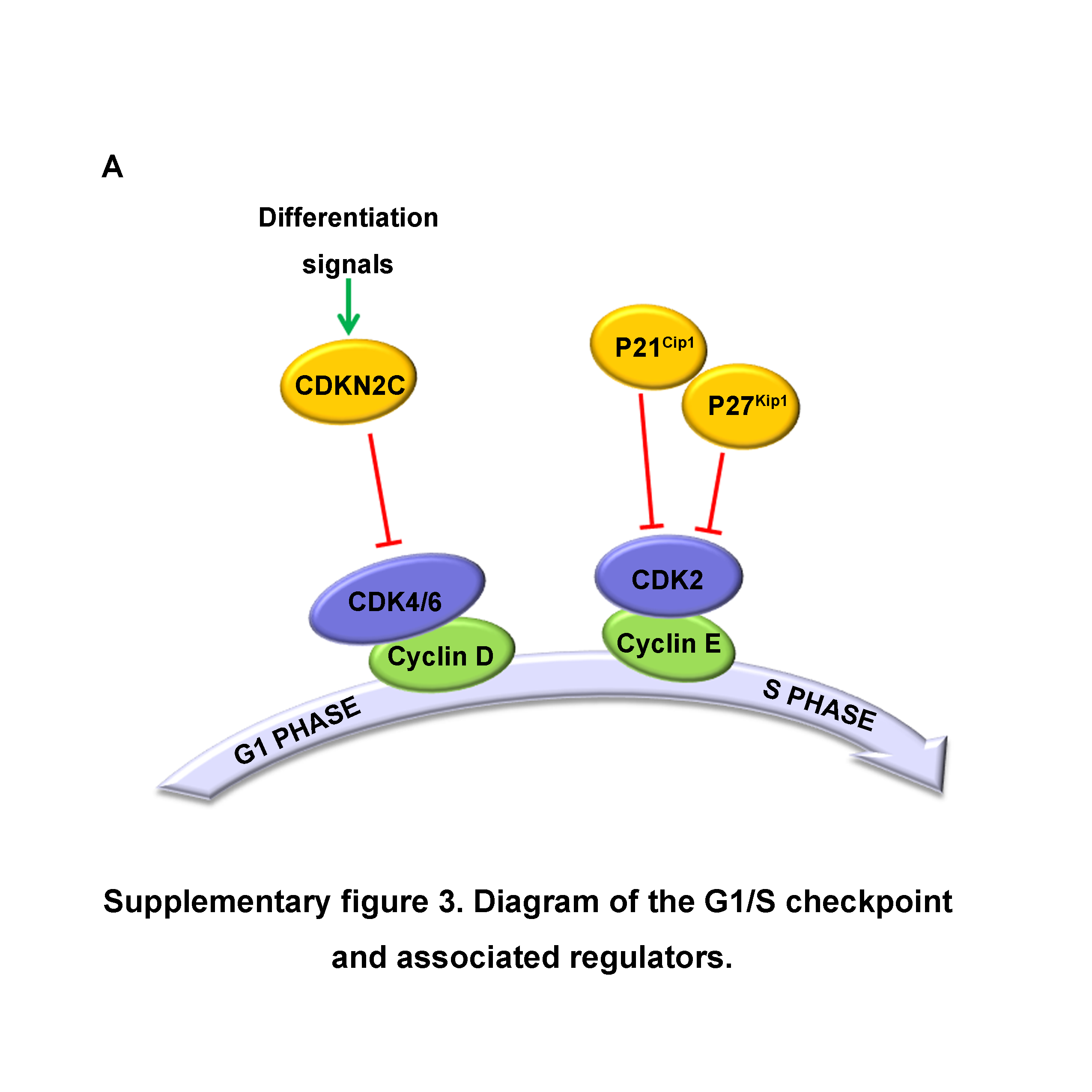

Supplement: Supplementary file 4 [file Image_3.TIF]

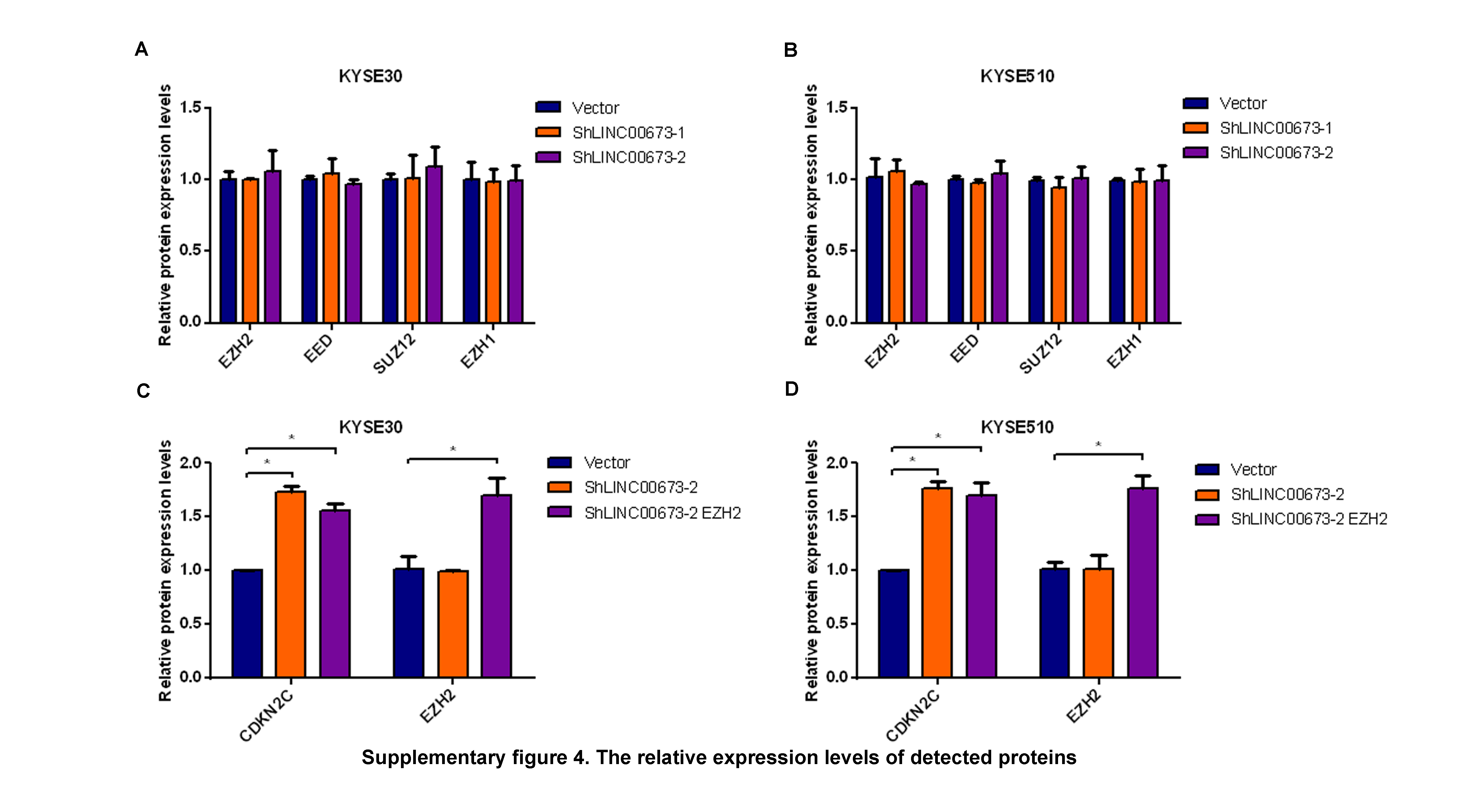

Supplement: Supplementary file 5 [file Image_4.TIF]
